# Supplementary figures and images for: The impact of signal variability on COVID-19 epidemic growth rate estimation from wastewater surveillance data
Source: PLoS One. 2025 May 28;20(5):e0322057. doi: 10.1371/journal.pone.0322057 (PMC12118983; doi:10.1371/journal.pone.0322057)

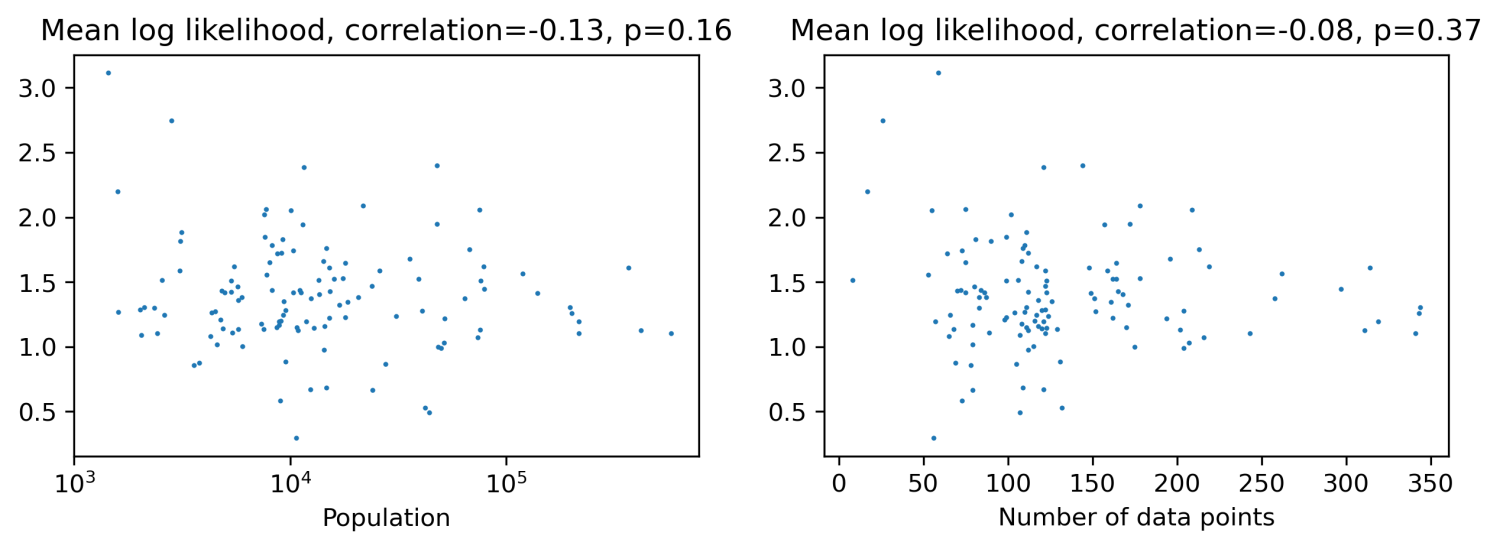

Supplement: S1 Fig — The log likelihood of model using the maximal likelihood parameter values is divided by the number of data points to give the mean log likelihood. Each point in these plots represents a WWTP (TIFF) [file pone.0322057.s001.tif]

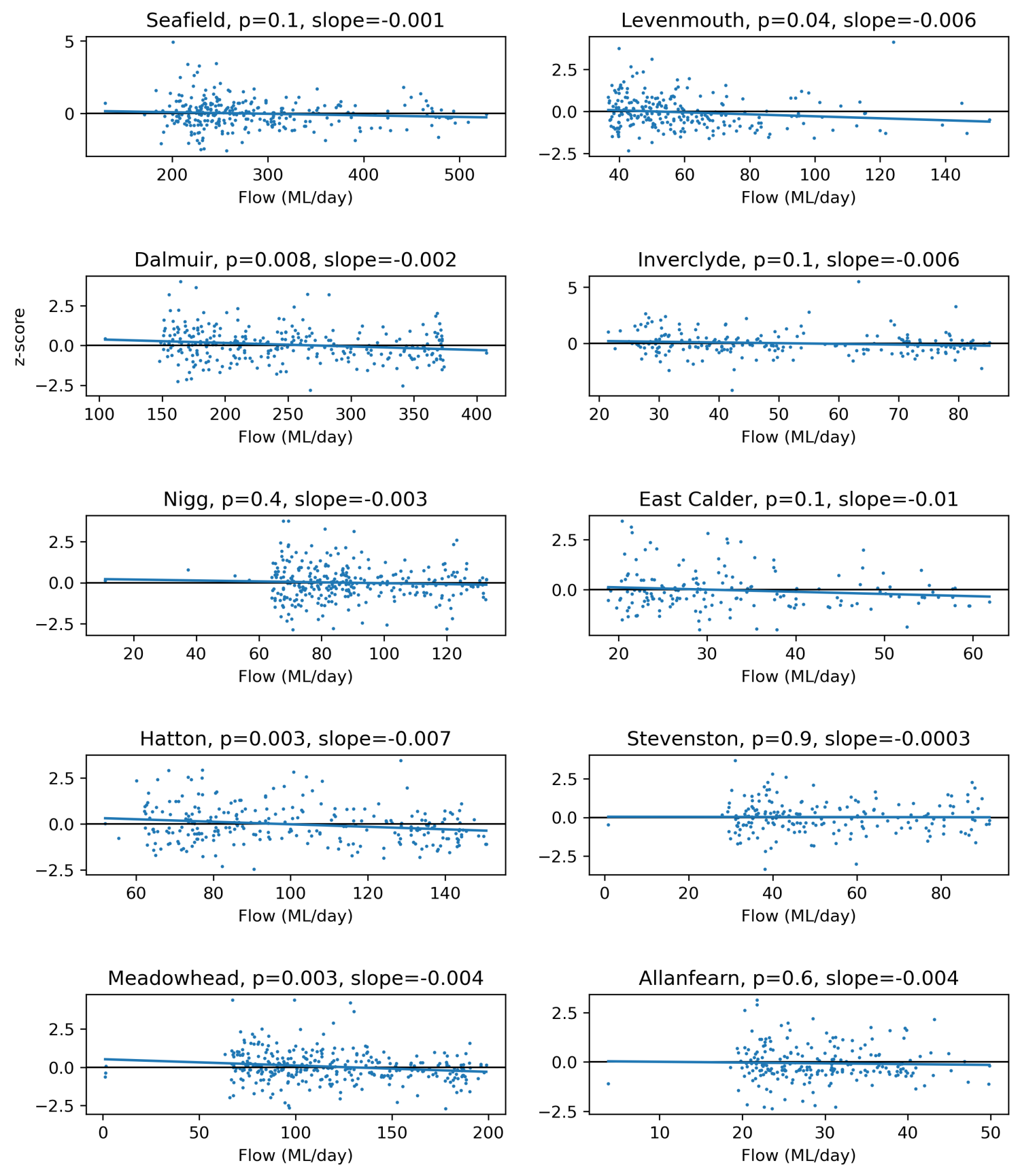

Supplement: S2 Fig — Each point in these plots represents a sample. The vertical axis shows the z-score for the sample with respect to the distribution that maximizes the model likelihood. (TIFF) [file pone.0322057.s002.tif]
