# Supplementary material for: The impact of signal variability on COVID-19 epidemic growth rate estimation from wastewater surveillance data
Source: PLoS One. 2025 May 28;20(5):e0322057. doi: 10.1371/journal.pone.0322057 (PMC12118983; doi:10.1371/journal.pone.0322057)
Supplement: Attachment — Submitted filename: Response to Reviewers.docx [file pone.0322057.s003.docx]

Ewan Colman

Bristol Medical School

Oakfield House

Oakfield Grove

Bristol, BS8 2BN

United Kingdom

28 February, 2025

PLOS One editorial office,

We would like to thank the editor and reviewers for their carful reading of our manuscript, and for their insightful comments and suggestions. Below is our response to each comment.

Sincerely,

Ewan Colman

~~~~~~~~~~~

Editor and reviewer comments are written as indented text.

**Editor**

1. Please ensure that your manuscript meets PLOS ONE's style requirements, including those for file naming.

We have reviewed the formatting guidelines and made the required changes

2. Please note that PLOS ONE has specific guidelines on code sharing for submissions in which author-generated code underpins the findings in the manuscript. In these cases, we expect all author-generated code to be made available without restrictions upon publication of the work. Please review our guidelines at https://journals.plos.org/plosone/s/materials-and-software-sharing#loc-sharing-code and ensure that your code is shared in a way that follows best practice and facilitates reproducibility and reuse.

Code is available at <https://github.com/EwanColman/Estimating-epidemic-growth-rate-from-wastwater-data>

3. We note that the grant information you provided in the ‘Funding Information’ and ‘Financial Disclosure’ sections do not match.

4. Thank you for stating in your Funding Statement:

“The first author was supported by the Wellcome Trust and the National COVID-19 Wastewater Epidemiology Surveillance Programme.”

Please provide an amended statement that declares *all* the funding or sources of support (whether external or internal to your organization) received during this study, as detailed online in our guide for authors at http://journals.plos.org/plosone/s/submit-now. Please also include the statement “There was no additional external funding received for this study.” in your updated Funding Statement.

Please include your amended Funding Statement within your cover letter. We will change the online submission form on your behalf.

The amended funding statement is as follows:

*EC was supported by Wellcome Trust (grant no. 209818/Z/17/Z) and the National COVID-19 Wastewater Epidemiology Surveillance Programme. RK received no specific funding for this work. The funders had no role in study design, data collection and analysis, decision to publish, or preparation of the manuscript. There was no additional external funding received for this study.*

5. Thank you for stating the following in the Acknowledgments Section of your manuscript:

“We are grateful to the Scottish Environmental Protection Agency, Scottish Water, and Public Health Scotland for making data for this study available. We thank our partners in the Scottish Government Wastewater Monitoring Data Integration Subgroup for their insightful feedback. Financial support was received from The Wellcome Trust and the National COVID-19 Wastewater Epidemiology Surveillance Programme.

We note that you have provided funding information that is currently declared in your Funding Statement. However, funding information should not appear in the Acknowledgments section or other areas of your manuscript. We will only publish funding information present in the Funding Statement section of the online submission form.

Please remove any funding-related text from the manuscript and let us know how you would like to update your Funding Statement. Currently, your Funding Statement reads as follows:

“The first author was supported by the Wellcome Trust and the National COVID-19 Wastewater Epidemiology Surveillance Programme.”

Please include your amended statements within your cover letter; we will change the online submission form on your behalf.

Please see the amended statement above. We have removed funders from the acknowledgements.

6. We note that you have indicated that there are restrictions to data sharing for this study. For studies involving human research participant data or other sensitive data, we encourage authors to share de-identified or anonymized data. However, when data cannot be publicly shared for ethical reasons, we allow authors to make their data sets available upon request. For information on unacceptable data access restrictions, please see http://journals.plos.org/plosone/s/data-availability#loc-unacceptable-data-access-restrictions.

Before we proceed with your manuscript, please address the following prompts:

a) If there are ethical or legal restrictions on sharing a de-identified data set, please explain them in detail (e.g., data contain potentially identifying or sensitive patient information, data are owned by a third-party organization, etc.) and who has imposed them (e.g., a Research Ethics Committee or Institutional Review Board, etc.). Please also provide contact information for a data access committee, ethics committee, or other institutional body to which data requests may be sent.

b) If there are no restrictions, please upload the minimal anonymized data set necessary to replicate your study findings to a stable, public repository and provide us with the relevant URLs, DOIs, or accession numbers. Please see http://www.bmj.com/content/340/bmj.c181.long for guidelines on how to de-identify and prepare clinical data for publication. For a list of recommended repositories, please see https://journals.plos.org/plosone/s/recommended-repositories. You also have the option of uploading the data as Supporting Information files, but we would recommend depositing data directly to a data repository if possible.

Please update your Data Availability statement in the submission form accordingly.

Hospital admissions data are now available in a de-identified anonymized form at <https://doi.org/10.6084/m9.figshare.28512479.v1>

7. We note that Figure 1 in your submission contain copyrighted images. All PLOS content is published under the Creative Commons Attribution License (CC BY 4.0), which means that the manuscript, images, and Supporting Information files will be freely available online, and any third party is permitted to access, download, copy, distribute, and use these materials in any way, even commercially, with proper attribution. For more information, see our copyright guidelines: http://journals.plos.org/plosone/s/licenses-and-copyright.

Figure 1 is an original image created by the authors.

8. We notice that your supplementary figures are uploaded with the file type 'Figure'. Please amend the file type to 'Supporting Information'. Please ensure that each Supporting Information file has a legend listed in the manuscript after the references list.

We have now uploaded the supplementary figures as 'Supporting Information'

**Reviewer 1**

*Only SARS-CoV-2 N1 was detected. The topic should be "The impact of signal variability on epidemic growth rate estimation of SARS-CoV-2 N1 from wastewater surveillance data".

Thank you for this suggestion. Our goal is to estimate the growth rate of COVID-19. The SARS-CoV-2 N1 gene count is the data we use to inform that estimate (not the thing we are aiming to estimate). We have changed the title to "*The impact of signal variability on COVID-19 epidemic growth rate estimation from wastewater surveillance data*"

*Please follow the PLOS ONE's abstract format.

Could the reviewer please be more specific in this request?

The abstract already meets the requirements for PLoS One: (a) Describe the main objectives of the study, (b) Explain how the study was done, and (c) Summarize the most important results and their significance. Moreover, it follows guidelines published by PLoS on how to write a good abstract <https://plos.org/resource/how-to-write-a-great-abstract/>

*The research gap is necessary for the last paragraph of the introduction.

We have added the following statement addressing the research gap. "*Within the existing literature, there are no studies that address the problem of inherent signal variability in designing a wastewater-based early warning system*."

*According to the methodology:

From February 2021 to the time of writing (February 2023), the Scottish Environment Protection Agency (SEPA) regularly took samples from wastewater treatment plants (WWTP) to detect fragments of SARS-Cov-2 virus RNA. Samples from sewerage influent were taken at 122 sampling locations across Scotland, approximately two to three days each week, depending on the location. *The study setting's population was wastewater from Scotland's WWTP. However, the sampling technique is crucial to ensure that the samples are appropriate for a representative of Scotland; please justify the samples from WWTP (the population) and the purposive selection or random selection for 122 sampling locations.

We do not claim that the data used is representative of any wider population. We only suggest that the data coming from a single WWTP is in some way representative of the population of its catchment area. However, to give some clarification we have added the following to the data section of the methods:

"*Combining the catchment areas of all 122 WWTPs covers the households of approximately 4500000 people, 82\% of the population of Scotland*."

If the data cannot be published, please add the statement, "Hospitalisation data were provided to us under a strict data sharing policy and can not be made public". But sampling technique is still necessary.

This is already included in the data availability statement. Regarding sampling, in the data section of the methods we have changed "*The hospital admission database includes information on individuals admitted to hospital in Scotland*" to "*The hospital admission database includes information on all individuals admitted to hospital in Scotland*"

*10 largest WWTPS shown in the result but not in the methodology, please add the sampling technique for 10 largest WWTPS.

We have added "*by catchment population*" each time we refer to the largest WWTP. Additionally, we have added to the data section of the methods "*The ten most populated catchments cover 47%* [of the population]."

*Please justify for data analysis details, such as the Likelihood of the underlying prevalence model and Epidemic trajectory model or other analysis.

With apologies to the reviewer, I do not understand what is being requested here. Please could you specify the part of the article to which it refers, and why it does not meet the criteria for publication?

*Please avoid using we and our, but use researchers and researchers instead.

My understanding is that it is not within the remit of a PLoS one reviewer to dictate the written style the article. We (the researchers) think that articles written in the first person are easier to follow.

*The conclusion could be more comprehensive based on how a strategic planner could improve the outcome or policies based on the study's results.

We address this with new material added to the discussion:

*We found on average that changes in the wastewater signal precede admissions by two days. Similar positive lags between wastewater and other indicators have been found previously, however, these results are based on retrospective analysis of time series data - they do not consider the accuracy of their predictions if they were to be deployed operationally in real time. Our methodology has been designed with the goal of operational use, that means providing information that can be acted upon in real time, potentially supporting resource management in hospitals (cancelling elective operations, increase staffing, stock up on oxygen). The 2-day lag found may provide more time, however, we warn that this is only an average; sometimes our method may produce false alarms, sometimes it may not detect change until after signal is seen in hospital admissions. Future work should explore how this method can be used in combination with other indicators.*

*It is suggested that limitations and recommendations for further studies be added.

This is addressed in the paragraph above, and in another new paragraph added to the discussion:

*While previous related literature has attempted to use environmental factors to explain signal variability, we have instead posited that most of the variability is caused by the non-dilution of particles containing large quantities of RNA. Future work could test this hypothesis experimentally. This could easily be done by installing two (or more) autosamplers at the same WWTP that are sampling at slightly different times (for example, offset by 30 minutes on a 1 hour sampling cycle) and comparing their outputs.*

**Reviewer 2**

Before a manuscript can be published major issues must be addressed by the author.

1. Abstract writing is not in accordance with the standard.

The abstract already meets the requirements for a PLoS One abstract: (a) Describe the main objectives of the study, (b) Explain how the study was done, and (c) Summarize the most important results and their significance. Moreover, it follows guidelines published by PLoS on how to write a good abstract <https://plos.org/resource/how-to-write-a-great-abstract/>

2. There are many similar studies, the author is asked to write a gap review to clarify the novelty of the research.

We have added the following statement addressing the research gap. "*Within the existing literature, there are no studies that address the problem of inherent signal variability in designing a wastewater-based early warning system*."

3. State of the art is not detailed enough, introduction is too short.

Could the reviewer please specify what details are required that are not included? It was our intention to keep the introduction short, only including necessary information while directing the reader to relevant literature for further details. If the reviewer would like to suggest other published papers that have similar aims to ours then we would be happy to include them.

4. The discussion is not deep enough and without enough comparisons.

We address this with new material added to the discussion:

*We found on average that changes in the wastewater signal precede admissions by two days. Similar positive lags between wastewater and other indicators have been found previously, however, these results are based on retrospective analysis of time series data - they do not consider the accuracy of their predictions if they were to be deployed operationally in real time. Our methodology has been designed with the goal of operational use, that means providing information that can be acted upon in real time, potentially supporting resource management in hospitals (cancelling elective operations, increase staffing, stock up on oxygen). The 2-day lag found may provide more time, however, we warn that this is only an average; sometimes our method may produce false alarms, sometimes it may not detect change until after signal is seen in hospital admissions. Future work should explore how this method can be used in combination with other indicators.*

5. The discussion presented is not only about modeling but the author must also discuss the environmental conditions/characteristics of wastewater and its relationship with epidemic growth.

We have added the following paragraph to the discussion to address this:

*While previous related literature has attempted to use environmental factors to explain signal variability, we have instead posited that most of the variability is caused by the non-dilution of particles containing large quantities of RNA. Future work could test this hypothesis experimentally. This could easily be done by installing two (or more) autosamplers at the same WWTP that are sampling at slightly different times (for example, offset by 30 minutes on a 1 hour sampling cycle) and comparing their outputs.*

6. Conclusions must be presented with numbers / research results.

The conclusion now starts with the following:

*We have contributed a novel procedure for estimating growth rates based on wastewater surveillance data and shown that it can provide an early warning for growing COVID-19 hospital admissions.*
