# Supplementary material for: The impact of signal variability on COVID-19 epidemic growth rate estimation from wastewater surveillance data
Source: PLoS One. 2025 May 28;20(5):e0322057. doi: 10.1371/journal.pone.0322057 (PMC12118983; doi:10.1371/journal.pone.0322057)
Supplement: Attachment — Submitted filename: Response_to_Reviewers_auresp_2.docx [file pone.0322057.s004.docx]

Ewan Colman

Bristol Medical School

Oakfield House

Oakfield Grove

Bristol, BS8 2BN

United Kingdom

11 March, 2025

PLOS One editorial office,

We would like to thank the editor and reviewer for their careful reading of our manuscript, and for their comments and suggestions. Below is our response to each comment.

Sincerely,

Ewan Colman

~~~~~~~~~~~

Editor and reviewer comments are written as indented text.

**Editor**

1. Please review your reference list to ensure that it is complete and correct. If you have cited papers that have been retracted, please include the rationale for doing so in the manuscript text, or remove these references and replace them with relevant current references. Any changes to the reference list should be mentioned in the rebuttal letter that accompanies your revised manuscript. If you need to cite a retracted article, indicate the article’s retracted status in the References list and also include a citation and full reference for the retraction notice.

We checked all references, identified one that needed correcting, and corrected it.

**Reviewer 1**

4. Have the authors made all data underlying the findings in their manuscript fully available?

The PLOS Data policy requires authors to make all data underlying the findings described in their manuscript fully available without restriction, with rare exception (please refer to the Data Availability Statement in the manuscript PDF file). The data should be provided as part of the manuscript or its supporting information, or deposited to a public repository. For example, in addition to summary statistics, the data points behind means, medians and variance measures should be available. If there are restrictions on publicly sharing data—e.g. participant privacy or use of data from a third party—those must be specified.

Reviewer #1: No

We have made data available in an anonymised de-identified format. The data statement we have provided is as follows

*RNA count from wastewater data are available from the Scottish Environmental Protection Agency, https://informatics.sepa.org.uk/RNAmonitoring/*

*Hospital admissions data in de-identified format are available from https://doi.org/10.6084/m9.figshare.28512479.v1*

*Treatment site catchment data are available in the code repository, https://github.com/EwanColman/Estimating-epidemic-growth-rate-from-wastwater-data*

*Comment 2 for Revision 1: Please add the abstract sections for the sub-headings of the introduction (background), methods, results, and conclusion. Data collection (sampling technique, such as purposive, convenience, or random sampling) and analysis (such as RT-qPCR) are necessary.

I do not think this is a requirement of PLOS one submissions. In fact, most PLOS one articles that I have seen do not follow this format. Perhaps the reviewer could provide a constructive reason why they think this format would improve the manuscript?

Similarly, the reviewer is asking for the method of measurement to be mentioned (we already say what is measured “*the number of copies of the N1*”). I do not agree with this change. Could they please clarify how this would improve the abstract?

To better describe the data used in this study we have changed “*The real-time growth rate of the SARS-CoV-2 prevalence is estimated at 121 wastewater sampling sites*” to “*The real-time growth rate of the SARS-CoV-2 prevalence is estimated at all 121 wastewater sampling sites*”

*Comment for Revision: Please add sampling techniques, such as random sampling, purposive sampling, or convenience sampling. Also, the data analysis using RT-qPCR. Please do not forget to adding the words, data collection and data analysis.

We addressed this comment in the previous response. We clarified that we had analysed data from *all* the available wastewater treatment sites. I would very much like to understand what remains unclear to the reviewer after reading our methods and the cited references, particularly ref. 25, and extensive description of the dataset used.

While you could call this a convenience sample, talking in that terminology may confuse readers as it implies that a statistic will be calculated on that sample and then extrapolated to a broader population. This does not describe the analysis we have performed.
